# Recall Efforts Successfully Increase Follow-Up for Cervical Cancer Screening Among Women With Human Papillomavirus in Honduras

**DOI:** 10.9745/GHSP-D-19-00404

**Published:** 2020-06-30

**Authors:** Kerry A. Thomson, Manuel Sandoval, Carolyn Bain, Francesca Holme, Pooja Bansil, Jacqueline Figueroa, Silvia de Sanjosé

**Affiliations:** a PATH, Sexual & Reproductive Health Program, Seattle, USA.; b Asociación Hondureña de Planificación de Familia, Tegucigalpa, Honduras.; c Secretary of Health, Tegucigalpa, Honduras.

## Abstract

Scaling up coverage of routine cervical screening in low-resource settings must be accompanied by efforts to retain women throughout the screening cascade and continuum of care, including adequate follow-up of abnormal results. The Scale-Up Project implemented human papillomavirus (HPV) testing for cervical cancer screening within public-sector health facilities in Honduras between 2015 and 2019. Women who were HPV-positive but did not have visually confirmed cervical lesions upon visual inspection with acetic acid (VIA-negative) were instructed to return to the health center after 1 year for repeat HPV testing. The current evaluation assessed the effectiveness of recall strategies to prompt women to return for retesting. Clinic staff placed reminder phone calls and followed up with short message service (SMS) or home visits, if needed. We summarized number of contacts, type of contacts, and time elapsed until return to the clinic, and used log-binomial regression to identify factors associated with return to the clinic. We identified 558 women who were initially HPV-positive VIA-negative from 8 clinics as needing repeat HPV testing 1 year later. Mean age was 43.2 years. Nearly all women (98.6%) were successfully contacted and 75.1% completed repeat HPV testing. The majority of contacts (65.4%) were phone calls, and nearly half of women who returned to the clinic (42.9%) did so after 1 contact. Mean days between contact and presentation at the clinic was 10.7 (standard deviation: 14.7). Women who required 3 or more contacts were 21% less likely to return for repeat HPV testing (prevalence ratio: 0.79; 95% confidence interval=0.69,0.90; *P*<.001) as compared to women who received only 1 contact. Reminder phone calls were highly successful at recalling women for HPV retesting in Honduras. This low-touch intervention should be included as part of standard follow-up to retain women throughout the continuum of cervical cancer screening and treatment.

## Abstract (precis)

A reminder phone call had a substantial impact on high rates of women returning for rescreening among those at high risk of developing cervical precancer. Scaling up routine cervical screening coverage must be accompanied by efforts to retain women throughout the screening cascade and continuum of care.

## Teaser

**Key Findings:**

- For most women at high risk of cervical precancer, a reminder phone call on the importance of rescreening for cervical cancer was sufficient to prompt women to return to the clinic for rescreening 1 year later.
- Women who needed to be contacted 3 or more times were significantly less likely to return to the clinic, suggesting that there will be diminishing returns to protracted tracing efforts per woman.

**Key Implications:**

- Cervical cancer screening is only effective when women with positive screening results are linked to treatment. Programs need to invest effort in robust follow-up systems for women with abnormal results at any step of the cervical cancer screening and treatment cascade.
- Program planners should build in reminder and recall strategies as part of a successful cervical cancer screening program.

## BACKGROUND

Cervical cancer is the fourth most common cancer affecting women worldwide, and an estimated 90% of deaths from cervical cancer occur in low- and middle-income countries (LMICs), highlighting the continued need for effective screening and treatment programs in these settings.^1^ The 2018 call for elimination of cervical cancer by World Health Organization (WHO) Director-General Dr. Tedros Adhanom Ghebreyesus is accompanied by ambitious targets for secondary prevention, including screening 70% of women at 35 years and 45 years of age with a high-precision screening test and treatment for 90% of women with detected cervical lesions.^2^ Scaling up coverage of routine cervical screening in LMICs must be accompanied by efforts to retain women throughout the screening cascade and continuum of care, including adequate follow-up of abnormal results and linkage to treatment.

Current WHO guidelines in settings with sufficient resources to implement human papillomavirus (HPV) testing include the option of following a primary HPV test with triage using visual inspection with acetic acid (VIA), treating women who both test positive for HPV and have visually confirmed cervical lesions, and repeating screening after 1 year for women who tested positive for HPV but do not have visible lesions.^3^ Rescreening women who are HPV-positive VIA-negative helps to address limitations in the combined sensitivity of HPV testing and VIA screening and offers a second opportunity to identify persistent HPV infections that are more likely to result in cervical precancer,^4^ while reducing potentially unnecessary treatment. The majority of women will clear their HPV infection within 1 to 2 years.^5^ However, women with persistent infection are at high risk for developing cervical lesions and warrant ongoing monitoring and/or treatment,^4^ especially in settings where a woman may only have 1 or 2 opportunities for primary screening in her lifetime.

A challenge of multi-step screening algorithms is opportunity for delays and loss to follow-up in between screening steps, especially when significant time elapses between contacts. In the Jujuy Demonstration Project in Argentina, women 30 years of age and older were first tested for HPV; women who tested positive for HPV underwent cytology. Of the 49,565 women tested, 67% were HPV-positive and had negative cytology; 70.1% of these women completed a repeat HPV test, although only 26% completed the retest within the recommended 12-18 month timeframe.^6^ Documented loss to follow-up from cervical cancer screening programs in low-resource settings is as high as 70%, although studies have mainly focused on attrition of women diagnosed with cervical precancer for whom treatment status is not known.^7^ There has been less focus on retention of screen-positive women who do not yet need treatment but do need continued surveillance for persistent HPV infection and development of cervical precancer. This subgroup of women, often overlooked in both program planning and reporting, are likely to increase in size as more countries adopt multistep screening algorithms.

Our objective was to evaluate the success of recall strategies to encourage women to return for follow-up ≥1 year after receiving HPV-positive VIA-negative screening results in public-sector health clinics in Honduras.

## INTRODUCTION OF HPV TESTING FOR CERVICAL CANCER SCREENING

The current evaluation was nested within the Scale-Up Project which was implemented in El Salvador, Guatemala, Honduras, and Nicaragua between 2014 and 2019. Project details have been described previously.^8^^,^^9^ In brief, in Honduras, PATH partnered with the Ministry of Health and a local nongovernmental organization, Honduras Association of Family Planning (ASHONPLAFA), to introduce HPV testing using *care*HPV (QIAGEN, Hilden, Germany), a signal-amplification batch diagnostic test for high-risk HPV DNA detection. A total of 44,314 women were tested for HPV across 3 departments (Copán, El Paraíso, and Region Metropolitana de Francisco Morazán, which includes the capitol city of Tegucigalpa).^8^

**FIGURE 1.**
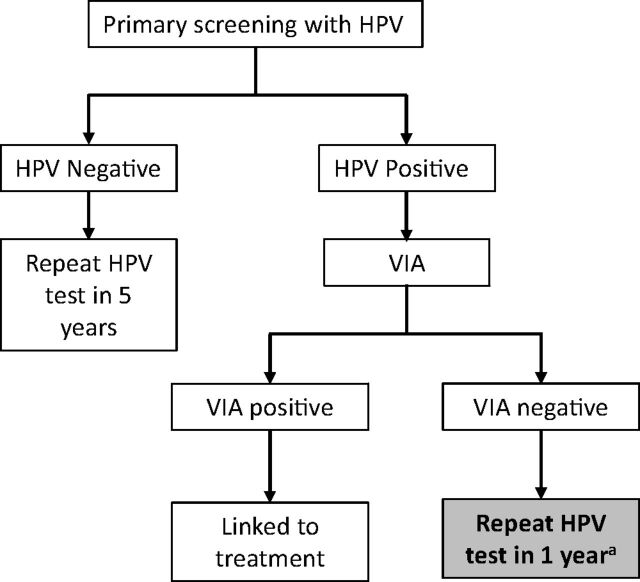
Cervical Cancer Screening Algorithm Followed in the Scale-Up Project, Honduras Abbreviations: HPV, human papillomavirus; VIA, visual inspection with acetic acid. ^a^ The current evaluation focuses on HPV-positive, VIA-negative women due for repeat HPV testing at 1 year.

Following WHO 2013 recommendations^3^ and 2015 Honduras guidelines^10^ for cervical cancer screening and treatment, women who tested positive for HPV were triaged using VIA to confirm the presence of lesions. Women who were HPV-positive VIA-positive were considered positive for cervical precancer and recommended for ablative treatment, if eligible, or more advanced treatment if needed. Women who were HPV-positive VIA-negative were considered negative for cervical precancer but counselled on the potential implications of persistent infection and instructed to return to the health center in approximately 1 year for repeat HPV testing (Figure 1). Initially, the Honduran health system did not actively track attendance at 1-year return visits for HPV-positive VIA-negative women; it was each individual woman’s responsibility to return for screening after 1 year. After anecdotal observation that very few women were returning for this follow-up visit, the current evaluation was designed to assess the success of various recall strategies to encourage women who were HPV-positive VIA-negative to return for follow-up ≥ 1 year after their initial screening result.

## IMPLEMENTATION OF RECALL STRATEGIES TO IMPROVE FOLLOW-UP

Women who were HPV-positive VIA-negative as part of the Scale-Up project after 2017 and had not yet spontaneously presented to the clinic for follow-up within 15 months of their initial screening date were traced between October 2018 and March 2019. For each family that receives care from public health clinics, a household health record is maintained, including demographics, contact information, dates of screening, and testing outcomes. Individual health visits (and dates) are recorded in a paper-based registry. In parallel, ASHONPLAFA maintained a Microsoft Excel registry of all HPV-positive VIA-negative women. The list of women’s names was provided to each clinic, where staff verified a woman’s initial screening result and date in the clinic-based paper register. As the first and primary outreach activity, staff attempted to contact each woman by phone. When successful contact was made, clinic staff reminded women of the need for HPV retesting and invited them to return to the clinic for a follow-up HPV test. If women could not be reached for conversation via phone, staff would attempt to send a SMS or conduct a home visit. If a woman still could not be reached, staff would attempt to call the alternate contact listed in the woman’s health record. Deidentified individual level data on the number and type(s) of contact and whether women completed retesting were recorded on paper forms and later entered into Excel. When women presented to the clinic for retesting, they were asked to report which recall method prompted them to return to the clinic. The algorithm for rescreening was consistent with that of the initial screening described above (Figure 1). Data on HPV test results, triage, and treatment outcomes were recorded in aggregate form at each clinic for women who had received outreach. The evaluation protocol was reviewed by PATH Research Determination Committee and was categorized as nonresearch. Data analysis was conducted using Stata (version 13.1, College Station, TX).^11^

## KEY FINDINGS

We included a total of 558 women who were HPV-positive VIA-negative and needed rescreening ≥1 year. Mean age among all women was 43.2 years (standard deviation [SD]: 9.6). Most women for whom age was available (70.8%) were between 30 and 50 years of age. Among women for whom parity was recorded, the majority (78.7%) of women had 2 or more children (Table 1).

**TABLE 1.** Demographic Characteristics of Women Who Were HPV-Positive VIA-Negative and Indicated for HPV Retesting After 1 Year, Honduras

**Characteristics**	
**All women**, No. (%)	558 (100)
**Clinic Location**	
Carrizal, No. (%)	77 (13.8)
Las Crucitas, No. (%)	48 (8.6)
San Benito, No. (%)	33 (5.9)
San Miguel, No. (%)	98 (17.6)
Alonzo Suazo, No. (%)	110 (19.7)
Villadela, No. (%)	59 (10.6)
Monterey, No. (%)	77 (13.8)
Pedregal, No. (%)	56 (10.0)
**Parity**	
No children, No. (%)	4 (0.7)
1 child, No. (%)	22 (3.9)
2 or more children , No. (%)	96 (17.2)
Not documented, No. (%)	436 (78.1)
**Age Category**, years	
< 30, No. (%)	4 (0.7)
30–39, No. (%)	162 (29.0)
40–49, No. (%)	122 (21.9)
50–59, No. (%)	90 (16.1)
≥ 60, No. (%)	23 (4.1)
Not documented, No. (%)	157 (28.1)
**Age**, mean (standard deviation)	43.2 (9.6)

**FIGURE 2.**
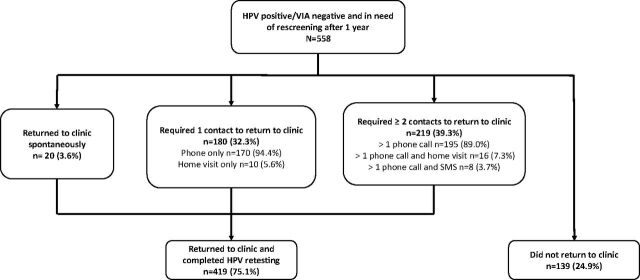
Overview of Recall Efforts to Encourage HPV-Positive VIA-Negative Women to Return for Clinic-Based HPV Testing ≥1 Year After Their First HPV-Positive Test Result Abbreviations: HPV, human papillomavirus; VIA, visual inspection with acetic acid.

A total of 419 women returned to the clinic for retesting, of which 20 women (3.6%) presented to the clinic spontaneously for retesting before being recalled by clinical staff and 399 (71.3%) required at least 1 contact before returning for retesting (Figure 2). Nearly half of women (45.1%) returned to the clinic after 1 contact, of whom the majority received 1 phone call (94.4%) and a small number of women received a home visit before a phone call (5.6%) because clinic staff were already in their neighborhood conducting other outreach activities. Slightly more than half of the women (54.9%) required more than 1 contact before presenting to the clinic for retesting; the majority of these women received phone calls only (89.0%) while a small proportion required a combination of phone call(s) followed by a home visit (7.3%) or phone call(s) followed by SMS (3.7%).

The maximum number of contact attempts for any woman was 8, and mean number of contacts received by women who returned to the clinic was 2.1 (SD: 1.5, Table 2). The average length of time elapsed between first contacting a woman and presenting to the clinic was 10.7 days. The majority of women (86.6%) reported that a phone call from clinic staff was the motivator that prompted them to return to the clinic and complete rescreening.

**TABLE 2.** Recall Methods Used To Encourage Repeat HPV Testing Among Women With Initial HPV-Positive VIA-Negative Screening Results, Honduras

	**Total** **N = 558**
Contacts received per woman, mean (SD) [range]	2.3 (1.5) [1–8]
Contacts received per woman who returned for 1-year follow-up (n=419^a^), mean (SD) [range]	2.1 (1.5) [0–8]
Phone calls/voicemails placed per woman, No. (%)	
1	211 (42.5)
2	118 (23.8)
3	86 (17.3)
4 or more	76 (15.3)
Missing	5 (1.0)
Days between first outreach and returning for 1-year follow-up (n=344^b^) , mean (SD) [range]	10.7 (14.7) [0–104)
Self-reported recall method that motivated clinic attendance (n=419) , No. (%)	
Telephone contact (phone call or text/SMS)	364 (86.9)
Home visit	25 (6.0)
None (presented spontaneously)	19 (4.5)
Voicemail	2 (0.5)
Not documented	9 (2.1)
Self-reported reasons for not returning for 1-year follow-up (n=139), No. (%)	
No reason specified	94 (67.6)
Repeat testing and follow-up happened at another clinic	25 (18.0)
Moved away from clinic area	9 (6.5)
Successfully contacted and declined	4 (2.9)
Cannot come due to work or personal reasons	3 (2.2)
Could not contact or locate	2 (1.4)
Pregnant	2 (1.4)

Abbreviations: HPV, human papillomavirus; VIA, visual inspection with acetic acid; SD, standard deviation.

a Denominator excludes women who did not return to the clinic.

b Denominator excludes women with missing information.

Nearly 87% of women reported that the clinic staff’s phone call motivated them to return to the clinic for rescreening.

Nearly one-quarter of women (24.9%) did not return for rescreening (Figure 2). The majority of these women (67.6%) were successfully contacted 1 or more times and did not report a specific reason for declining rescreening but also did not return to the health clinic before the end of the evaluation period (Table 2). A small proportion of women could not be contacted (1.4%), had moved (6.5%), reported barriers to clinic attendance (2.2%), cited pregnancy status as a reason for not wanting rescreening (1.4%), or reported refusal to return to the clinic (2.9%). Eighteen percent of women reported that they had already been rescreened (and if needed, treated) at a different health facility.

Table 3 presents differences between women who did and did not return to the clinic for rescreening. Women who required 3 or more contacts were 21% less likely to return for rescreening (prevalence ratio [PR]: 0.79; 95% confidence interval [CI]=0.69,0.90; *P*<.001) as compared to women who received only 1 contact. Women with 2 or more children were 9% less likely to return to the clinic (PR: 0.91; 95% CI=0.85,0.97; *P*=.003) as compared to women who reported no children. There were some statistically significant differences in the success of recall efforts across clinic sites; women from San Miguel, Villa Adela, Monterey, and Pedregal clinic sites were 30% more likely to complete screening as compared to women in Carrizal (Table 3).

**TABLE 3.** Factors Associated With Completion of HPV Retesting Among Women With Initial HPV-Positive VIA-Negative Screening Results, Honduras, N=544^a^

	**Did Not Return to Clinic** **n=139**	**Returned to Clinic for HPV Retesting** **n=415**		
	**No. (%) [95%CI]**	**No. (%) [95%CI]**	**Prevalence Ratio (95% CI)**	***P* Value**
Number of contacts^b^				
1 contact	46 (20.4) [15.6,26.1]	180 (79.7) [73.9,84.4]	Ref	—
2 contacts	22 (18.6) [12.6,26.8]	96 (81.4) [73.2,87.4]	1.02 (0.92,1.14)	.70
≥ 3 contacts	71 (37.2) [30.6,44.3]	120 (62.8) [55.7,69.4]	0.79 (0.69,0.90)	<.001
Parity				
No children	0 (0)	4 (100.0)	Ref	—
1 child	2 (9.5) [2.3,32.0]	19 (90.5) [68.0,97.7]	0.91 (0.79,1.04)	.16
2 or more children	9 (9.6) [5.0,17.5]	85 (90.4) [82.5,95.0]	0.91 (0.85,0.97)	.003
Not documented	128 (29.4) [25.3,33.9]	307 (70.6) [66.1,74.7]		—
Clinic				
Carrizal	26 (33.8) [24.0,45.1]	51 (66.2) [54.9,76.0]	Ref	—
Las Crucitas	25 (52.1) [38.0,65.9]	23 (47.9) [34.1,62.0]	0.72 (0.52,1.01)	.06
San Benito	6 (18.8) [8.5,36.3]	26 (81.2) [63.7,91.5]	1.22 (0.97,1.55)	.08
San Miguel	14 (14.3) [8.6,22.8]	84 (85.7) [77.2,91.4]	1.29 (1.08,1.54)	.005
Alonzo Suazo	41 (37.3) [28.7,46.7]	69 (62.7) [53.3,71.3]	0.95 (0.76,1.17)	.62
Villadela	7 (12.1) [5.8,23.4]	51 (87.9) [76.6,94.2]	1.32 (1.10,1.60)	.003
Monterey	11 (14.7) [8.3,24.7]	64 (85.3) [75.3,91.7]	1.29 (1.08,1.55)	.007
Pedregal	9 (16.1) [8.5,28.3]	47 (83.9) [71.7,91.5]	1.27 (1.04,1.54)	.02
Age category, years				
30–39	12 (7.4) [4.2,12.6]	150 (92.6) [87.4,95.8]	Ref	—
40–49	6 (4.9) [2.2,10.6]	116 (95.1) [89.4,97.8]	1.03 (0.97,1.09)	.38
50–59	9 (10.0) [5.3,18.2]	81 (90.0) [81.8,94.7]	0.97 (0.90,1.05)	.50
>60	2 (8.7) [2.1,29.6]	21 (91.3) [70.4,97.9]	0.99 (0.86,1.13)	.84
Not documented	110 (70.1) [62.4,76.7]	47 (29.9) [23.3,37.6]	—	—

Abbreviations: CI, confidence interval; HPV, human papillomavirus; VIA, visual inspection with acetic acid.

a Excludes women < 30 years of age (n=4).

b Excludes women who returned spontaneously (n=20).

## IMPLICATIONS FOR CERVICAL CANCER SCREENING PROGRAMS

Our study describes the substantial impact that simple recall efforts, primarily phone calls, had on encouraging women to complete follow-up for cervical cancer surveillance and successfully engaging them in rescreening. Although the Tegucigalpa population is slightly mobile, most women could be contacted ≥ 1 year after their initial screening visit. In the majority of cases, phone calls alone were sufficient to recall women, nearly half of the women who returned to the clinic did so after 1 contact, and most women returned within 2 weeks of being contacted. These findings suggest that for most women a reminder on the importance of rescreening was sufficient to overcome any potential barriers to clinic attendance and adherence to follow-up appointments that are commonly reported in the literature.^12^^–^^15^ Women who needed to be contacted 3 or more times were significantly less likely to return to the clinic, suggesting that there will be diminishing returns to protracted tracing efforts per woman. These results underscore the value of building in recall strategies as part of a successful cervical cancer screening program and demonstrate that a simple recall phone call can have a big impact on retention.

These results underscore the value of building in recall strategies as part of a successful cervical cancer screening program.

Although screening approaches will continue to vary widely across settings, it is critical for programs to invest effort in robust follow-up systems for women with abnormal results at any step of the cervical cancer screening cascade.^4^ Monitoring and retaining women throughout the continuum of cervical cancer screening is a key component to reduce morbidity and mortality associated with cervical cancer. Earlier work to assess the cost-effectiveness of various screening approaches in Latin American has demonstrated the importance of adequate follow-up of abnormal screening.^16^ A mathematical model based on various screening scenarios in Colombia estimated that 50% coverage with 100% follow-up reduced cervical cancer mortality by 21.3% more than a scenario with 100% coverage and 50% follow-up.^17^

Although we did not document the HPV test result for all women who were traced and returned to the clinic for retesting, registry data from the 8 clinics serving the same patient population indicate that among a sample of 298 women who initially were HPV-positive VIA-negative, 36% tested positive for HPV ≥ 1 year later. This evidence confirms the need for continued surveillance of this subgroup over time. Among a cohort of Dutch women who were HPV-positive and had negative cytology, 56.6% were HPV-positive when retested an average of 10 months later.^18^ Women who test positive on 2 consecutive HPV tests may be candidates for treatment, especially in low-resource settings where engagement with the health system may be limited. Another option that takes into consideration high rates of persistent HPV infection and the challenges recalling women for retesting is a “test-and-treat” approach, wherein treatment is offered to all women who are HPV-positive without a triage step. A recent study in Papua New Guinea using cytology as the reference standard concluded that treating all women who are HPV-positive resulted in appropriate treatment of 92% of women with high-grade disease and 13% overtreatment, as compared to a combined algorithm of HPV testing followed by VIA for triage which resulted in 45.5% appropriate treatment and 3.7% overtreatment.^19^ El Salvador has adopted this approach; all women who are HPV-positive receive VIA to confirm eligibility for cryotherapy, but treatment is not conditional on visual confirmation of lesions.^20^

This intervention required only moderate appropriation of staff time and use of clinic phones, but we did not track detailed costs associated with adding recall efforts to the cervical cancer screening approach in Honduras. ASHONPLAFA and Ministry of Health personnel championed the intervention and emphasized the importance of recalling women and encouraged persistent tracing. A costing study of cervical cancer screening in South Africa found that tracing activities reduced 12- and 24-month loss to follow-up by nearly 30% and successfully engaging a woman for follow-up at 24 months was twice as expensive as at 12 months. However, at the time the previous study was conducted (2005), this target population did not have high mobile phone ownership, thus requiring community health workers to make more resource-intensive home visits compared to the clinic-based efforts described here.^7^

This intervention required only moderate appropriation of staff time and use of clinic phones.

Our evaluation has demonstrated that the addition of a low-touch intervention in Honduras captured 75% of women indicated for retesting. Phone calls were successful and sufficient to reach the majority of women, even in a setting where mobile phone numbers frequently change. Few women required follow-up through alternative methods including SMS. Although a manual process of individual phone calls was successful in the context of this particular project, it is important to consider alternate and more automated communication channels when replicating and possibly scaling up a similar intervention to reach a larger number of women. Health information systems such as the open source District Health Information Software 2 (DHIS2) or canSCREEN developed by the Australian VCS Foundation specific to cancer surveillance have the option to flag patients who are overdue for screening and auto-generate recall reminders by SMS or phone call.

### Limitations

Women residing in the urban and periurban areas included in our evaluation live in close proximity to the health facilities that contacted them. However, women’s ability to access services may still be encumbered by the security situation and community violence in Honduras. Thus, our results may have limited generalizability to women residing in other settings, such as rural areas, where access barriers are likely different.

The current evaluation did not include a comparison group, although less than 4% of the women included in our screening population returned to the clinic for retesting on their own before receiving a reminder from clinic staff, suggesting that a large number of women would not have initiated retesting in the absence of recall efforts. Literature on the impact of reminder and recall strategies for cervical cancer screening from LMICs is limited. An evaluation of an automated reminder system integrated into the national health information system in Denmark found that prompting general practitioners to remind their female patients to return for 12-month follow-up of abnormal cytology reduced loss to follow-up by 48%.^21^ In low-resource settings that do not yet have robust digital health information systems, mobile health interventions, including 1- or 2-way texting platforms^22^ and apps, are currently under evaluation for retaining women in the cervical cancer cascade and may offer a more efficient, systematic, and cost-effective approach than individualized phone calls, especially when delivered at scale as part of a national screening program. In health systems where specific appointment dates are set, reminder phone calls placed in advance of a target date can also be considered.

## CONCLUSION

Surveillance and follow-up of abnormal screening results is paramount to the success of an effective cervical cancer screening program. As countries scale up screening and treatment efforts, a reminder and recall system, such as the low-touch phone reminders described here, should be included as part of a comprehensive cervical cancer control strategy.

## Notes

**En español**

**Alto seguimiento de mujeres VPH positivas en el tamizado de cáncer de cuello uterino en Honduras tras contacto recordatorio**

**Principales Conclusiones**

- Para la mayoría de las mujeres a alto riesgo de lesiones precancerosas del cuello uterino, un recordatorio telefónico sobre la importancia de la visita de seguimiento fue suficiente para una alta asistencia al año de la primera visita.
- El retorno en visitas de seguimiento fue muy inferior en las mujeres que necesitaron más de tres contactos telefónicos podría cuestionar la viabilidad de esfuerzos adicionales en la captación de mujeres reticentes.

**Principales Implicaciones**

- El tamizaje cervical solo es efectivo si se aborda adecuadamente el manejo de las mujeres con resultados anormales del tamizaje. Es necesario que los programas de tamizaje dispongan de un sistema de seguimiento robusto para conseguir un adecuado seguimiento en toda la cascada de eventos que se origina en el tamizaje y tratamiento.
- Los programas de tamizaje deben de incluir recordatorios y estrategias de captación de las visitas de seguimiento para conseguir que los programas de tamizaje sean exitosos.

**RESUMEN**

Parar retener a las mujeres que participan en programas de tamizaje del cáncer de cuello uterino en países de bajos recursos económicos es necesario considerar esfuerzos que garantizen la continuidad de la atención que incluya un adecuado seguimiento de resultados anormales. El Proyecto Scale-Up implementó la prueba del virus del papiloma humano (VPH) para el tamizaje del cáncer de cuello uterino en las instalaciones de salud del sector público en Honduras entre 2015 y 2019. Las mujeres que eran positivas para el VPH pero no tenían lesiones cervicales confirmadas visualmente recibieron instrucciones de regresar al centro de salud después de 1año para repetir la prueba de VPH. La evaluación actual evaluó la efectividad de las estrategias de recuerdo para hacer que las mujeres regresen para volver a realizar la prueba. El personal de las clínicas participantes realizo llamadas telefónicas recordatorias y seguimiento con SMS. En casos necesario se realizaron visitas a domicilio. Resumimos el número de contactos, el tipo de contactos y el tiempo transcurrido hasta el regreso a la clínica, y utilizamos la regresión log-binomial para identificar los factores asociados con el regreso a la clínica. Inicialmente, se identificaron 558 mujeres con una prueba VPH positiva pero que habían resultado tener prueba concomitante de IVAA negativa y que según recomendaciones, necesitaban repetir la prueba de VPH después de 1 año. La edad promedio fue de 43.2 años. La mayoría de las mujeres (98.6%) fueron contactadas con éxito y el 75.1% fueron sometidas a una prueba de seguimiento de VPH. La mayoría de los contactos (65.4%) se realizaron a través de llamadas telefónicas y casi la mitad de las mujeres que regresaron a la clínica (42.9%) lo hicieron después de un primer contacto. La media de días entre el contacto y la presentación en la clínica fue de 10.7 (desviación estandard: 14.7). Las mujeres que requirieron tres o más intentos de contacto tenían un 21% menos de probabilidades de regresar para repetir la prueba del VPH (ratio de prevalencia: 0.79; intervalo de confianza del 95%=0.69,0.90, *P*<.001) en comparación con las mujeres que recibieron un solo contacto. Se concluye que en Honduras las llamadas telefónicas recordatorias fueron muy satisfactorias para realizar un seguimiento adecuado y re-analizar a las mujeres para un posible persistencia del VPH. Se recomienda la introducción de esta intervención sencilla y de bajo coste como parte del seguimiento estandarizado para retener a las mujeres durante la cascada de detección, manejo y tratamiento del cáncer de cuello uterino.

**Peer Reviewed**

**First published online:** May 8, 2020
